# A case of recurrent gallstone ileus within 1 week post enterolithotomy

**DOI:** 10.1093/jscr/rjac057

**Published:** 2022-03-04

**Authors:** Emily Doole

**Affiliations:** Department of General Surgery, University Hospital Geelong, Barwon Health, Geelong, Victoria 3220, Australia

## Abstract

Gallstone ileus (GSI) is a rare pathology, affecting 0.3–0.5% of people with cholelithiasis and accounting for 0.1% of mechanical bowel obstructions. It carries a high mortality of 12–27%. The rate of recurrence following a first episode of GSI is relatively high at 5–20%. Very early recurrence, within the first week, poses several challenges in both diagnosis and management. This case describes an 84-year-old woman who presented with a mechanical bowel obstruction secondary to gallstone impacted in the distal jejunum. This was managed operatively and the patient progressed well. The patient developed an early recurrent gallstone ileus, confirmed on computed tomography (CT) on Day 7 postoperatively. This was initially mistaken for a postoperative ileus. Early recurrent gallstone ileus can easily be mistaken for more common postoperative complications. Given its high mortality, consideration should be given to early investigation with CT to rule out recurrent GSI.

## INTRODUCTION

Cholelithiasis is a common pathology affecting an estimated of 10–15% of people in the developed countries. [1] Gallstone ileus (GSI) as a complication of cholelithiasis is, however, a relatively rare phenomenon. GSI affects $\sim$0.3–0.5% of patients with gallstones and is responsible for only 0.1% of mechanical bowel obstructions. [2] It carries a high mortality of 12–27%. [2] The mechanism behind the development of GSI is the formation of an internal biliary fistula created between the gallbladder and the gastrointestinal tract. This fistula is most common between the gallbladder and the duodenum (75–83% of cases). [3]

The incidence of recurrence following a first episode GSI is relatively high, estimated between 5 and 20%. [4] There are many case reports documenting patients who have suffered multiple recurrences, placing them at repeated high risk of morbidity and mortality. Most recurrences will occur within 6 months of initial presentation: $\sim$62% within the first 6 weeks and 82% within the first 6 months. [4, 5]

There are two main surgical approaches to the management of GSI. The first is simple enterolithotomy, where the objective of the operation is simply to relieve the emergent issue of obstruction. [5] The second is a single stage procedure where enterolithotomy is performed as well as repair of the biliary fistula. [5] Simple enterolithotomy carries a mortality rate of 4.2%, whereas a single stage procedure carries a substantially higher mortality rate of 22.2%. Consensus tends to favour simple enterolithotomy as first line, with an option of delayed fistula closure. [1].

## CASE PRESENTATION

An 84-year-old woman presented to the emergency department with a 3-day history of nausea, abdominal pain and distension. She had witnessed faeculent vomit in the emergency department. Her abdomen was visibly distended with generalized tenderness but no focal or generalized signs of peritonism.

The patient had a medical history of atrial fibrillation anticoagulated on apixaban, type 2 diabetes mellitus, a previous ischaemic stroke and mild cognitive impairment. She had no known history of gallstones.

**Figure 1:**
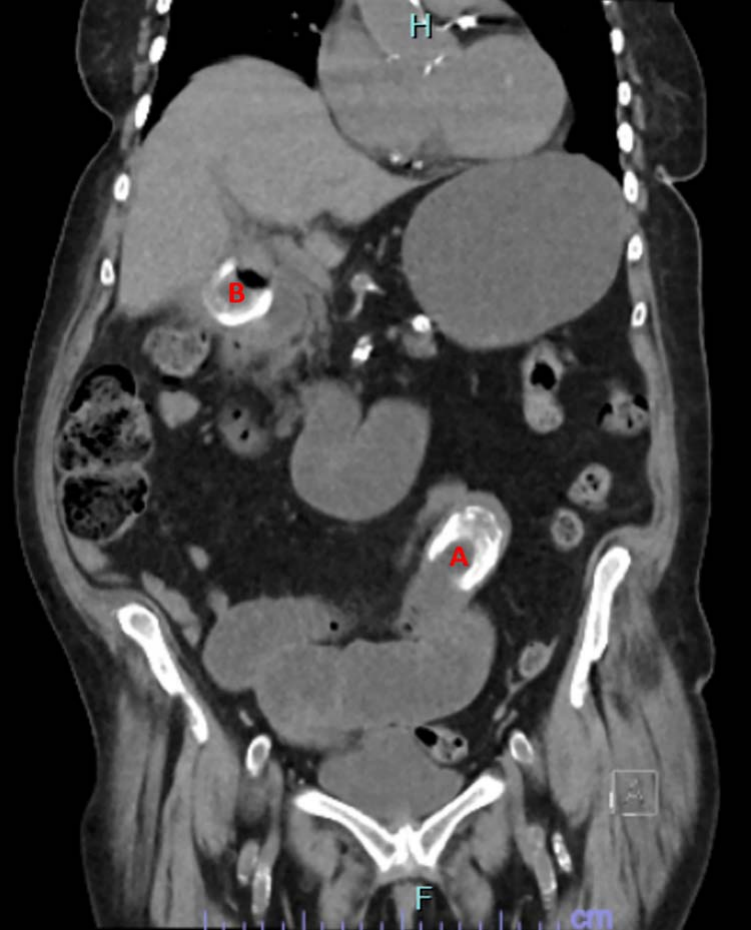
CT image showing obstructing gallstone (**A**) and gallstone in cholecystoduodenal fistula traversing duodenal wall (**B**).

Her investigations found mildly elevated inflammatory markers, with a white cell count of 11.7 and a C-reactive protein of 75. A computed tomography (CT) abdomen and pelvis was performed which found a high-grade bowel obstruction secondary to a large gallstone lodged in the distal jejunum (Fig. 1). A second gallstone was identified located in the gallbladder, with the possibility raised that the gallstone was partially traversing the duodenal wall.

On Day 1 post presentation, the patient was taken for an exploratory laparotomy where the transition point was identified in the mid-jejunum. The bowel wall at the site of obstruction was compromised but viable. An enterotomy was performed at the site of the stone and the stone was successfully removed. At the time of the surgery her right upper quadrant was noted to have extensive adhesions and the gallbladder itself was unable to be visualized.

The patient progressed well in the immediate postoperative period. She passed flatus and opened her bowels on Day 1 postoperatively, and her nasogastric tube was able to be removed on Day 2. She was tolerating a light diet by Day 3.

On Day 6 postoperatively, the patient developed nausea and vomiting. Her bowels opened normally that day. Her abdomen was mildly distended and appropriately tender around her incision site. Her nausea and vomiting were attributed to a postoperative ileus. The patient developed a postoperative hypoactive delirium, making her assessment more challenging.

By Day 7 the patient’s symptoms had continued to worsen. A repeat CT abdomen and pelvis was arranged to rule out postoperative complications. Unexpectedly, the CT showed that the patient had developed a recurrent GSI, with the gallstone previously located in the gallbladder now lodged in the distal jejunum causing a high-grade small bowel obstruction. The patient returned to theatre for a repeat laparotomy. An enterotomy was performed and the stone successfully removed. The operation was made more challenging due to the presence of extensive fresh adhesions. Once again, the right upper quadrant was not entered and the gallbladder not visualized.

The patient progressed well postoperatively with no further complications. She was able to be transferred to a facility for inpatient rehabilitation and at the time of writing remains well.

## DISCUSSION

Recurrent GSI poses several difficulties in both diagnosis and management. This is especially apparent in cases of early recurrence. Diagnosis may be hindered or delayed due to the symptoms mimicking other more common postoperative complications. The management is also challenging because of the hostile nature of entering the abdomen within the first week following a laparotomy. The risk of inadvertent enterotomy or other complications makes this repeat operation more technically challenging and higher risk.

In a review of the available literature, five previous case reports were identified which also described recurrent GSI within the first week postoperatively. [6–10] These patients all had known remaining gallstones at the time of first operation; however, due to the high risk of a single stage procedure, all underwent simple enterolithotomy. Several of the cases also commented on adhesions or inflammatory changes in the right upper quadrant making accessing the gallbladder and the remaining gallstones challenging.

In a condition carrying such a high mortality rate, such as recurrent GSI, prompt diagnosis and commencement of an appropriate management plan are critical. Despite the rarity of recurrent GSI, the risk of a missed diagnosis is high, so consideration should be given to a CT to differentiate recurrent GSI from postoperative ileus.

## CONCLUSION

GSI is a rare condition, associated with a high mortality rate. It poses a number of challenges, in particular deciding on the best operative management to address the emergent obstruction while considering the risk of recurrent ileus. Recurrent GSI is even more uncommon and diagnosis is made difficult in early recurrence by its tendency to mimic other, more common, postoperative complications. If a patient has known remaining gallstones following a GSI and simple enterolithotomy and they develop symptoms of ileus or obstruction in the postoperative period, imaging (for example CT) should be considered early to reduce the risk of a missed recurrence.

## DECLARATIONS

E.D. collected case information and was directly involved in patient care, collated patient information and formulated the review of literature, case review and discussion. The corresponding author attests that all listed authors meet authorship criteria and that no others meeting the criteria have been omitted.
